# Integration of selective sweeps across the sheep genome: understanding the relationship between production and adaptation traits

**DOI:** 10.1186/s12711-024-00910-w

**Published:** 2024-05-21

**Authors:** Pablo A. S. Fonseca, Aroa Suárez-Vega, Juan J. Arranz, Beatriz Gutiérrez-Gil

**Affiliations:** Departamento de Producción Animal, Facultad de Veterinaria, Universidad de León, Campus de Vegazana S/N, 24071 León, Spain

## Abstract

**Background:**

Livestock populations are under constant selective pressure for higher productivity levels for different selective purposes. This pressure results in the selection of animals with unique adaptive and production traits. The study of genomic regions associated with these unique characteristics has the potential to improve biological knowledge regarding the adaptive process and how it is connected to production levels and resilience, which is the ability of an animal to adapt to stress or an imbalance in homeostasis. Sheep is a species that has been subjected to several natural and artificial selective pressures during its history, resulting in a highly specialized species for production and adaptation to challenging environments. Here, the data from multiple studies that aim at mapping selective sweeps across the sheep genome associated with production and adaptation traits were integrated to identify confirmed selective sweeps (CSS).

**Results:**

In total, 37 studies were used to identify 518 CSS across the sheep genome, which were classified as production (147 prodCSS) and adaptation (219 adapCSS) CSS based on the frequency of each type of associated study. The genes within the CSS were associated with relevant biological processes for adaptation and production. For example, for adapCSS, the associated genes were related to the control of seasonality, circadian rhythm, and thermoregulation. On the other hand, genes associated with prodCSS were related to the control of feeding behaviour, reproduction, and cellular differentiation. In addition, genes harbouring both prodCSS and adapCSS showed an interesting association with lipid metabolism, suggesting a potential role of this process in the regulation of pleiotropic effects between these classes of traits.

**Conclusions:**

The findings of this study contribute to a deeper understanding of the genetic link between productivity and adaptability in sheep breeds. This information may provide insights into the genetic mechanisms that underlie undesirable genetic correlations between these two groups of traits and pave the way for a better understanding of resilience as a positive ability to respond to environmental stressors, where the negative effects on production level are minimized.

**Supplementary Information:**

The online version contains supplementary material available at 10.1186/s12711-024-00910-w.

## Background

The sheep (*Ovis aries*) is a domestic species known for its diversity and adaptation potential, thriving in extreme agroecological conditions. It was one of the first species to be domesticated in the Middle East approximately 9000–11,000 years before present, originated from wild Asian mouflon species (*Ovis orientalis*) [1–4]. Initially, used for meat production, sheep were later selected for milk and wool production approximately 4000–5000 years ago, leading to the existence of over 1400 sheep breeds today. During domestication, genetic phenomena such as the “bottleneck” effect occurred, leading to a decrease in reproductive individuals and in their genetic variability [5]. However, at the same time, the process of splitting the population could have led to an increase in the total variability between populations. Artificial selection acts on the target population to create animals that meet human needs, resulting in different breeds of the same species. This process involves changes in the frequency of loci responsible for controlling phenotypes, impacting not only causal mutations but also nearby genetic markers due to linkage disequilibrium [6–9].

A signature of selection refers to a genome region with increased frequency of a specific allele in a population due to its functional importance during a selection process [10]. Natural and artificial selection drive the appearance of these signatures, which are characterized by reduced diversity not only directly in the affected mutations but also in nearby regions [11, 12]. Various statistical methods are available to identify signatures of selection, including comparing allelic frequencies between related breeds using F_ST_ or other associated statistics, analysing regions with low diversity or haplotypes, and identifying extreme allelic frequency patterns in a population [10, 13–15]. Genetic markers, particularly single nucleotide polymorphisms (SNPs), have been used in many studies to identify signatures of selection in domestic species such as cows and sheep [10, 11, 16, 17]. Following the identification of signatures, the next step typically involves evaluating genes within the identified regions to identify functional candidate genes that explain the effects of signatures of selection. Advancements in genomic technologies, such as commercial medium-density SNP chips and the reduced cost of next-generation sequencing (NGS), have facilitated studies on signatures of selection in sheep. Such studies have focused on specialized dairy or meat production breeds, the fat tail phenotype, and disease resistance [18–22]. More recently, studies have emphasized signatures of selection associated with the adaptation of sheep to specific environments such as high altitudes or heat stress [23, 24]. Another important characteristic of the livestock sector that has received increasing attention in recent years is animal resilience [25]. Resilience is the ability of animals to be minimally affected and/or rapidly respond to a disturbance of their health, welfare or productivity status [26]. Understanding the biological mechanisms that are associated with different adaptation responses might be useful in identifying candidate genomic regions and genes to be used for selecting more resilient animals.

Understanding the genetic basis of adaptation and preserving sheep genetic resources are crucial for genetic improvement, conservation, and sustainable livestock production [23, 27–29]. In spite of the advancements brought by recent studies on identifying signatures of selection in domestic animals, there are still inherent challenges. These include combining multiple types of analysis and statistical methods to eliminate false-positive results, difficulty in linking signatures of selection to a specific phenotype since the analysis is solely based on genotypic data, and the challenge of identifying the gene and causal mutation responsible for the signature of selection. To address these challenges, it is crucial to select the populations to be included in the study, combine various types of analyses within a single study, and compare results obtained from different studies conducted on different breeds. Studying the biological function of genes in a selection region helps identify genes linked to traits affected by selection. This allows inferring the potential phenotype associated with that selection signature across specific traits.

In light of the above, the objectives of this study were: (1) to perform a systematic review of studies reporting signatures of selection across the ovine genome associated with production and adaptation traits; (2) to combine the information from multiple studies to identify confirmed signatures of selection (CSS); (3) to integrate the information of positional candidate genes within CSS coordinates to identify potential functional profiles associated individually with productivity or adaptability; and (4) to compare functional candidate genes for productivity and adaptability to identify putative candidate genes underlying pleiotropic effects that may exist between these two classes of traits.

## Methods

### Selection of studies

PubMed (https://pubmed.ncbi.nlm.nih.gov/) was used as search engine, where the following searches were made: “selection signatures sheep”, “selection signatures sheep production”, “selection signatures sheep ecoregions”, and “selective sweep sheep adaptation”. In general, all the articles where the term “adaptation” was present in the title of the description of the population analyzed, were considered as related to adaptation, whereas those that analyzed populations bred for production traits (meat, milk, and wool) were considered as production. Fat tail-related studies, where the classification was unclear, were considered to be “adaptation” related studies.

### Identification of confirmed signatures of selection

The remaining articles were examined to retrieve the genomic coordinates for the reported selective sweeps. The NCBI Remap (https://www.ncbi.nlm.nih.gov/genome/tools/remap) tool was used to convert all the coordinates to the version ARS-UI_Ramb_v2.0 of the ovine reference genome. For studies where the selective sweep location was reported as the coordinate of a single marker, a 250-kb interval downstream and upstream from this coordinate was calculated for the remapping step. Those studies that did not report the complete coordinates from the detected selective sweeps were also removed at this step. In addition, those studies where the selective sweeps were mapped based on an old version of the ovine reference genome for which conversion of coordinates to the ARS-UI_Ramb_v2.0 reference genome was not possible were also removed. A summary of the workflow applied to select the articles that composed the final sample is presented in Additional file 1 Figure S1.

The next step was the definition of the CSS that would be studied. For this purpose, the selective sweeps that were reported by three or more different studies and overlapped within the same genomic region were classified as CSS. The region comprising the CSS from all the overlapping studies was defined as the flanking region (smallest and largest genomic coordinate). In addition, for each CSS, the proportion of production and adaptation studies supporting the CSS was calculated. Subsequently, those CSS composed of more than 60% selective sweeps derived from production studies were called production CSS (prodCSS). In comparison, the CSS that comprised more than 70% of adaptation selective sweeps were called adaptation CSS (adapCSS).

### Annotation of positional candidate genes and quantitative trait loci (QTL) mapped within the CSS coordinates

The positional candidate genes and quantitative trait loci (QTL) previously reported within the defined CSS were annotated using the GALLO package in R v.4.2.0 [30]. The gtf file used for gene annotation corresponding to the ARS-UI_Ramb_v2.0 of the ovine genome was obtained from NCBI. For the QTL annotation, the gff file from Sheep QTLdb corresponding to the ARS-UI_Ramb_v2.0 of the ovine genome was used. The GALLO package was also used to perform a QTL enrichment analysis for each trait annotated within the CSS flanking interval in the Sheep QTLdb using a genome-wide approach. The enriched QTL were defined based on a false-discovery rate (FDR) < 0.05 and a total number of QTL reported in the Sheep QTLdb larger than 1.

In addition, an enrichment analysis for gene ontology (GO) terms for the three categories available (biological process (BP), molecular function (MF), and cellular component (CC) was performed using the R package gprofiler2 [31]. In order to better understand the functional profile of the genes associated with prodCSS and adapCSS, the enrichment analyses for GO terms were performed individually for the genes annotated exclusively within prodCSS, for the genes annotated exclusively within adapCSS, and for the genes shared between both CSS classes. In addition, the R package rutils [32] was used to reduce the redundancy of GO terms through the go_reduce() function, where the Wang measure was selected to identify similar GO terms with a 0.7 threshold. The child GO terms assigned to the same GO parental terms were grouped into the same class, and the smallest p-value from the child terms was assigned to the parental term. The relationship between the positional candidate genes and the enriched GO terms related to production and adaptation was investigated using a network approach where the R packages igraph [33] and visNetwork [34] were used to identify hub genes of these networks, classified as those genes with the betweenness above the 90% quantile. The betweenness of a node in a network is defined as the number of shortest paths that pass through that node. Consequently, this metric can be used as a signal of the relevance of a gene in an interaction network. Similarly to the QTL enrichment analysis, the enriched GO terms and KEGG pathways were defined based on an FDR < 0.05 threshold. However, only GO terms with less than 1000 genes assigned to them in the gprofiler2 database were considered to avoid broad terms that might not be informative.

## Results

### Selected studies for the identification of confirmed signatures of selection

In total, 43 articles were retrieved from PubMed. However, five articles were excluded based on different criteria. Detailed information regarding all 43 articles and the exclusion criteria are shown in Additional file 2 Table S1. Among the 43 articles retained for the identification of CSS, 23 articles were classified as adaptation-related studies, and 15 articles were defined as production-related articles (Table 1). The coordinates of each selective sweep reported in the 38 selected studies are available in Additional file 3 Table S2 and were used for identifying CSS considering the Oar_rambouillet_v2.0 sheep reference genome.

**Table 1:** Studies retained for identification of confirmed selective sweeps associated with adaptation and production across the sheep genome

Study code	Study	Pubmed ID	Breeds	Trait	Selective sweep metric
A1	[35]	32071365	Djallonké and Burkina-Sahel	Regulation of oxidative and metabolic stress, thermotolerance, and trypanotolerance	iHS, XP-EHH, and nSL
A2	[36]	25074012	Romney and Perendale	Resistance/susceptibility gastrointestinal nematodes	Fst
A3	[37]	26523558	Laticauda, Cyprus fat tail, Alpagota, Altamurana, Appenninic, Bergamasca, Biellese, Delle Langhe, Gentile, Fabrianese, Istrian Pramenka, Massese, Sambucana, Sarda, and Sopravissana	Thin tail vs fat tail	Fst
A4	[38]	26555032	Barki	Adaptation to hot arid environment	Fst and iHS
A5	[39]	27809776	Mongolian, Duolang, Luobu, Buyinbuluk, Jinzhong, Tan, Yuxi fat-tailed, Small-tailed Han, Large-tailed Han, Wadi, Luzhong Shandi, Sishui Fur, and Hu	Short fat-tailed sheep vs sheeps from different ecoregions	Fst and pooled heterozygosity
A6	[40]	29247174	Egyptian fat-tail	Fat tail	di, Rsb, and iHS
A7	[41]	30883840	Baluchi, Lori-Bakhtiari, and Zel	Adaptation to three different Iranian climatic regions	XP-EHH and Fst
A8	[42]	31001329	Tibetan, Mongolian, Altay, Hu and Duolang	Comparison between selected breeds and wild ancestors of domestic sheep (Asian mouflon, Ovis orientalis)	Fst and Hp
A9	[19]	31699027	Djallonke and Sahelian	Resistance to Haemonchosis and resilience to animal trypanosomiasis	HomSI
A10	[43]	31365135	Valais Red, Valais Blacknose, Bundner Oberlander, Engadine Red, Saaser Mutten, Swiss Black Brown Mountain, Swiss Mirror, Swiss White Alpine	Local adaptation	Fst and di
A11	[44]	31119684	Blackhead Somali, Arsi-Bale, Horro, Adilo, and Menz	Adaptation to mid to high-altitude areas, cool sub-alpine environments, and arid and semi-arid areas	Fst
A12	[45]	32039702	Buubei, Lezgin, Karachaev, Karakul, Tuva, Edilbai, Romanov (GROUP1), Russian Longhaired, Altai Mountain, Groznensk, Salsk, Volgograd, Krasnoyarsk, Baikal, and Kulundin (GROUP2)	Acclimation (GROUP1 vs GROUP2)	DCMS and hapFLK
A13	[46]	31615414	Katahdin, St. Croix, Dorper	Resistant vs susceptible breeds for *Haemonchus contortus*	Fst
A14	[47]	33110149	Lowland Tibetan, Langkazi Tibetan, Jiangzi Tibetan, Gangba Tibetan, Huoba Tibetan, Duoma Tibetan, Awang Tibetan, Linzhou	High‑altitude adaptation	Fst and ZHp
A15	[48]	31199810	Bonga, Doyogena, Gesses, Kido, Loya, Shubi, Kefis, Arabo, Adane, Molale, Gafera, Huri, Naimi, Najdi, Omani, Barbaresca, Laticauda, Libyan, Hammari, Kabashi, Sardinian, Comisana, Sidaoun	Tail fat deposition	Rsb and Fst
A16	[49]	32365888	Hetian, Karakul, Yabuyi, Hu, and Wadi	Adaptation to extremely dry or humid environments	Fst and XP-EHH
A17	[50]	34,349,777	Tan Sheep, Oula, Zeku, and Black Tibetan	Thin tail vs Fat tail	ROH
A18	[51]	33708236	Alpine merino, chinese merino, aohan fine-wool, qinghai fine-wool	Plateau adaptability	Fst and Tajima’s D
A19	[52]	33107621	Tuva and Baikal	Environmental stresses in temperature	H1, H12, Tajima’s D, Pi, and Fst
A20	[53]	33501931	Adane, Arabo, Bonga, Doyogena, Kefis, Segentu, Gesses, Kido, Loya, Menz, Shubi Gemo, and Washera	Adaptation to altitude and four environmental variables	Fst and PBS
A21	[22]	33715983	Fat-tailed Barbarine, Queue fine de l'Ouest, and Noire de Thibar	Fat-tailed vs thin-tailed	ROH and Fst
A22	[54]	35843722	East Friesian milk, Dorper, Texel, South African mutton merino, Black Suffolk, Australian white, Mongolian, Lanzhou large-tailed, Altay, large-tailed Han, and Asiatic mouflons	Domestication vs improvement	Fst
A23	[55]	36685923	Merino, Meatmaster, Afrino, South African Merino, South African Mutton Merino, Damara, Ronderib, Afrikaner, Nguni	Merino vs non-merino breeds	iHS
P1	[56]	24004915	Altamurana	Milk yield	Comparison of the allele frequency at each marker between top and worse milk yielders
P2	[57]	24788864	Chios, Sakiz, Churra, Ojalada, Comisana, Australian Poll Merino, East Friesian Brown, Finnsheep, Milk Lacaune, Meat Lacaune	Dairy production	Fst and ObsHtz
P3	[58]	29115919	Australian Industry Merino, Australian Merino, Australian Poll Merino, and Spanish Churra	Wool production	Fst and ObsHtz
P4	[59]	28463982	Belclare, Beltex, Charollais, Suffolk, Texel and Vendeen	Selective pressure for meat production	ROH and Fst
P5	[60]	28187715	Assaf and Awassi	Body size, fat tail, horn shape, wool type, coat pigmentation, Seasonality, Adaptation to Middle-Eastern climate, milk production, prolificacy	Fst
P6	[61]	29566643	Sasi Ardi and Latxa	Regions underlying local adaptation, regions related to a long traditional dairy selection pressure in Latxa sheep, and regions experiencing the specific effect of the modern genetic improvement program established for the Latxa breed	Pooled heterozygosity H, Tajima’s D, and Fst
P7	[62]	31708969	Spanish Merino, Australian Merino, Chinese Merino, Sopravissana, and Gentile di Puglia	Wool production	Fst and ROH
P8	[63]	31379930	Kazakh	Number of thoracic vertebrae	Fst and Hp
P9	[64]	33445473	Bamei Mutton	Litter size	Fst
P10	[65]	34002409	Luzhong mutton	Litter size	ROH
P11	[21]	35681887	Zel and Lori Bakhtiari	Fat-tailed vs thin-tailed	Fst and XP-EHH
P12	[66]	35099062	South African Mutton Merino, Australian Merino and Chinese Merino	Wool production	Fst, iHS, and XP-EHH
P13	[67]	35428998	Ethiopian Bonga and Menz	Top EBVs from Community-based breeding programs (CBBPs) vs non-CBBPs	Fst, LR, XP-EHH
P14	[68]	35681809	Valachian	Original Valachian vs Improved Valachian sheep	ROH
P15	[69]	36244020	Pishan and Bashbay	Litter size	Fst and pi ratio

*iHS* integrated Haplotype Score, *XP-EHH* cross-population extended haplotype homozygosity, *nSL* number of segregating sites by length, *Fst* fixation index, *di* function of pairwise Fst between breed one and the remaining breeds, *Hp* pooled homozygosity, *HomSI* homozygosity stretch identifier, *DCMS* de-correlated composite of multiple signals, *hapFLK* haplotype-based FLK, *ZHp* Z-transformed pooled homozygosity, *ROH* runs of homozygosity, *H1* haplotype homozygosity, *H12* haplotype homozygosity statistics, *PBS* population-branch statistic, *ObsHtz* observed heterozygosity

### Confirmed signatures of selection for adaptation and production

The 529 CSS identified are available in Additional file 4 Table S3. Among these CSS, 213 adaptation CSS (adapCSS) and 172 production CSS (prodCSS) were selected based on the percentage of adaptation and production studies validating the CSS (see Additional file 4 Table S3), where a large number of CSS showed a ratio between 60 and 70% for both categories (see Additional file 5 Figure S2). In total, 4318 and 3092 genes were annotated within adapCSS and prodCSS, respectively (see Additional file 6 Table S4). In addition, 1851 genes were shared between adapCSS and prodCSS. However, it is not possible to disregard the potential functionality of these genes for both production- and adaptation-related traits.

### Correspondence with QTL effects and functional analysis for positional candidate genes within confirmed signatures of selection

**Fig. 1:**
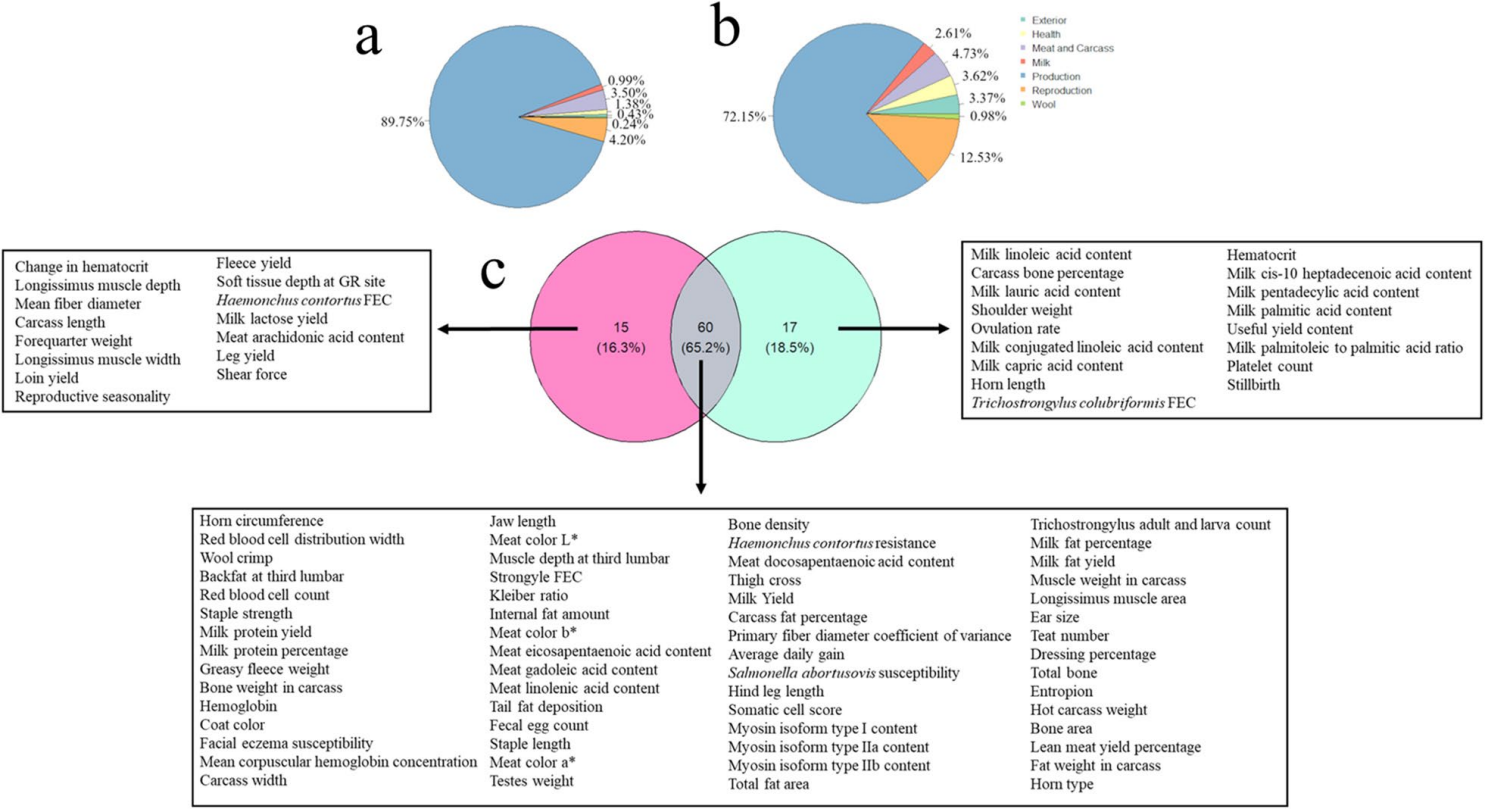
Results of the annotation of quantitative trait loci (QTL) for the confirmed selective sweeps (CSS). **a** Pie plot showing the percentage of each QTL trait type annotated within the coordinates of the CSS composed by more than 60% of production studies (prodCSS); **b** Pie plot showing the percentage of each QTL trait type annotated within the coordinates of the CSS composed by more than 60% of adaptation studies (adapCSS); and **c** Venn diagram describing the number of enriched QTL trait terms identified exclusively and shared for the prodCSS (in pink) and adapCSS (in green). The associated enriched QTL trait terms are highlighted in the text boxes

The annotation of QTL for adapCSS and prodCSS resulted in similar patterns. For both, the production QTL class was the most frequent, representing 89.75% and 72.15% of all the QTL annotated for prodCSS and adapCSS, respectively (Fig. 1a, b). In addition, slight increases in the percentages of QTL classes related to the reproduction, wool, health, and exterior classes were observed for adapCSS when compared to prodCSS. In total, 75 and 77 QTL were enriched for prodCSS and adapCSS, respectively (see Additional file 7 Table S5). The majority of these QTL (60 QTL) were shared between prodCSS and adapCSS (Fig. 1c). Among the QTL enriched exclusively for prodCSS (15 QTL), it is relevant to highlight the relatively large number of QTL related to wool (mean fibre diameter and fleece yield) and meat (longissimus muscle depth, longissimus muscle width, forequarter weight, loin yield, soft tissue depth at the GR site, carcass length, meat arachidonic acid content, leg yield and shear force). Regarding the QTL exclusively enriched for adapCSS, some that are related to adaptability, such as haematocrit, platelet count, ovulation rate, horn length, *Trichostrongylus colubriformis* FEC, and stillbirth, are worth mentioning. In addition, it is relevant to mention the number of milk fatty acid-related QTL observed as exclusively enriched for adapCSS (8 out 17 QTL): linoleic acid, lauric acid, conjugated linoleic acid, capric acid, cis-10 heptadecenoic acid, pentadecylic acid, palmitic acid, and palmitoleic acid. In spite of the identification of milk fatty-acids QTL as enriched exclusively on adapCSS, it is important to reinforce the importance of these QTL for production traits. Following this expectation, the enriched QTL terms shared between adapCSS and prodCSS comprise a combination of all QTL classes (Fig. 1c). However, it is important to mention the enrichment of QTL terms related to morphological traits associated with the selection process to which different sheep breeds were subjected, such as coat colour, horn circumference, horn type, teat number, jaw length, hind leg length, and ear size. In addition, several QTL related to fat deposition in different deposits were identified as enriched for both adapCSS and prodCSS, such as backfat at the third lumbar vertebra, tail fat deposition, internal fat amount, carcass fat percentage, total fat area, and fat weight in the carcass.

**Fig. 2:**
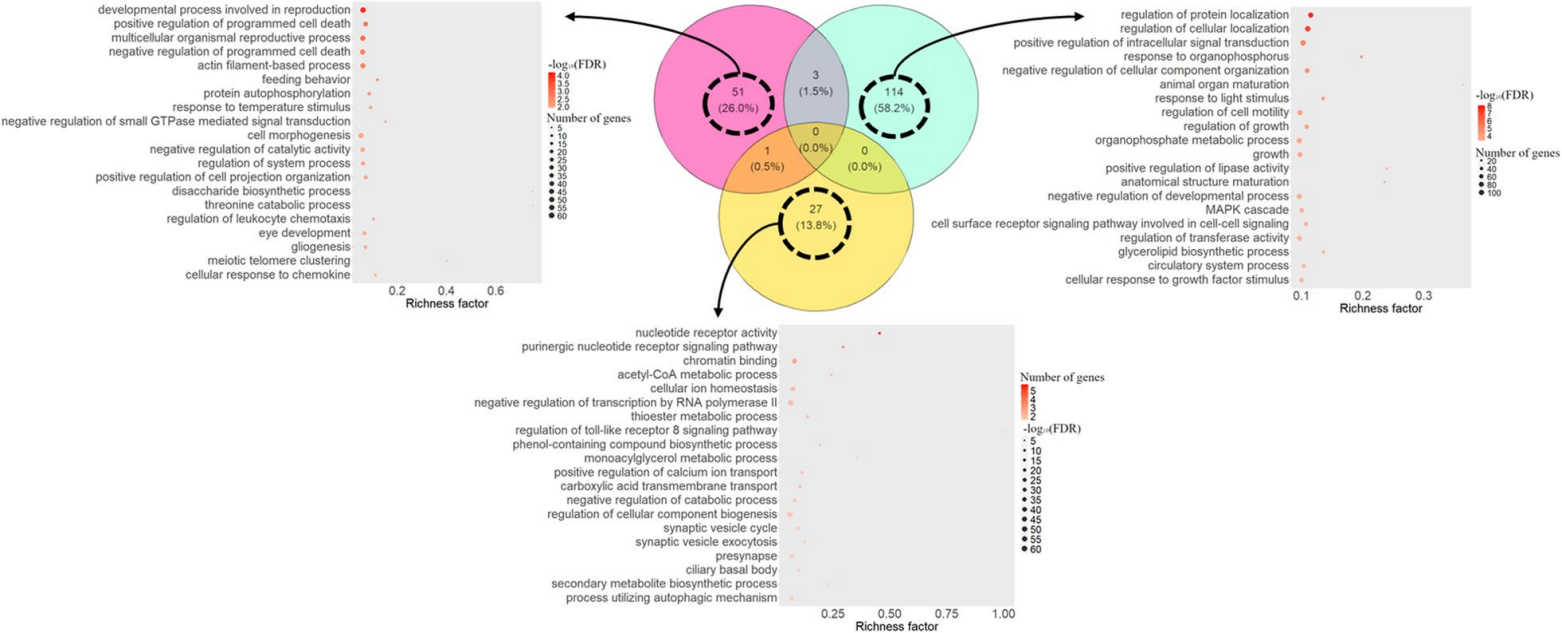
Number of enriched gene ontology (GO) terms identified for genes harboring exclusively CSS composed by more than 60% of production studies (prodCSS) in pink, genes harboring exclusively CSS composed by more than 60% of adaptation studies (adapCSS) in green, and harboring both prodCSS and adapCSS in gold. The top 10 enriched GO terms for each group are shown in the bubble plots, where the area of the bubbles corresponds to the number of associated genes and the color indicates the p-value scale (the darker colour, the smaller the false-discoveryrRate adjusted p-value). The richness factor shown in the x-axis is the ratio between the number of genes annotated in the current study associated with a specific GO term divided by the total number of genes associated with this specific term in the database

Regarding the enriched GO terms, 51 and 114 terms were identified exclusively for the genes that harboured only prodCSS or adapCSS, respectively (Fig. 2). In addition, 27 enriched GO terms were exclusively enriched for the list of genes harbouring both adapCSS and prodCSS. The complete list of enriched GO terms is available in Additional file 8 Table S6. Among the top 20 most enriched terms for the genes harbouring prodCSS, we highlight GO terms related to reproduction, response to temperature stimulus, response to chemokines and feeding behaviour (Fig. 2). For genes harbouring exclusively adapCSS, among the top 20 most enriched GO terms are terms related to cellular organization, signal transduction, response to light stimulus, organ maturation and growth (Fig. 2). In addition, a relevant number of enriched terms associated with lipid metabolism and adaptative thermogenesis were observed (see Additional file 9 Figure S3). The analysis of the network composed of these terms and the associated genes indicated the connection of these terms by functionally candidate genes, such as *ELOVL3*, *SCD*, *IP6K1*, *FLCN*, *IL18*, and *FFAR4*. Regarding the enrichment results for those genes harbouring both prodCSS and adapCSS, the metabolism of acetyl-CoA and monoacylglycerol, as well as the signalling of purinergic receptors, stand out as candidate processes for adaptation and production in sheep.

**Fig. 3:**
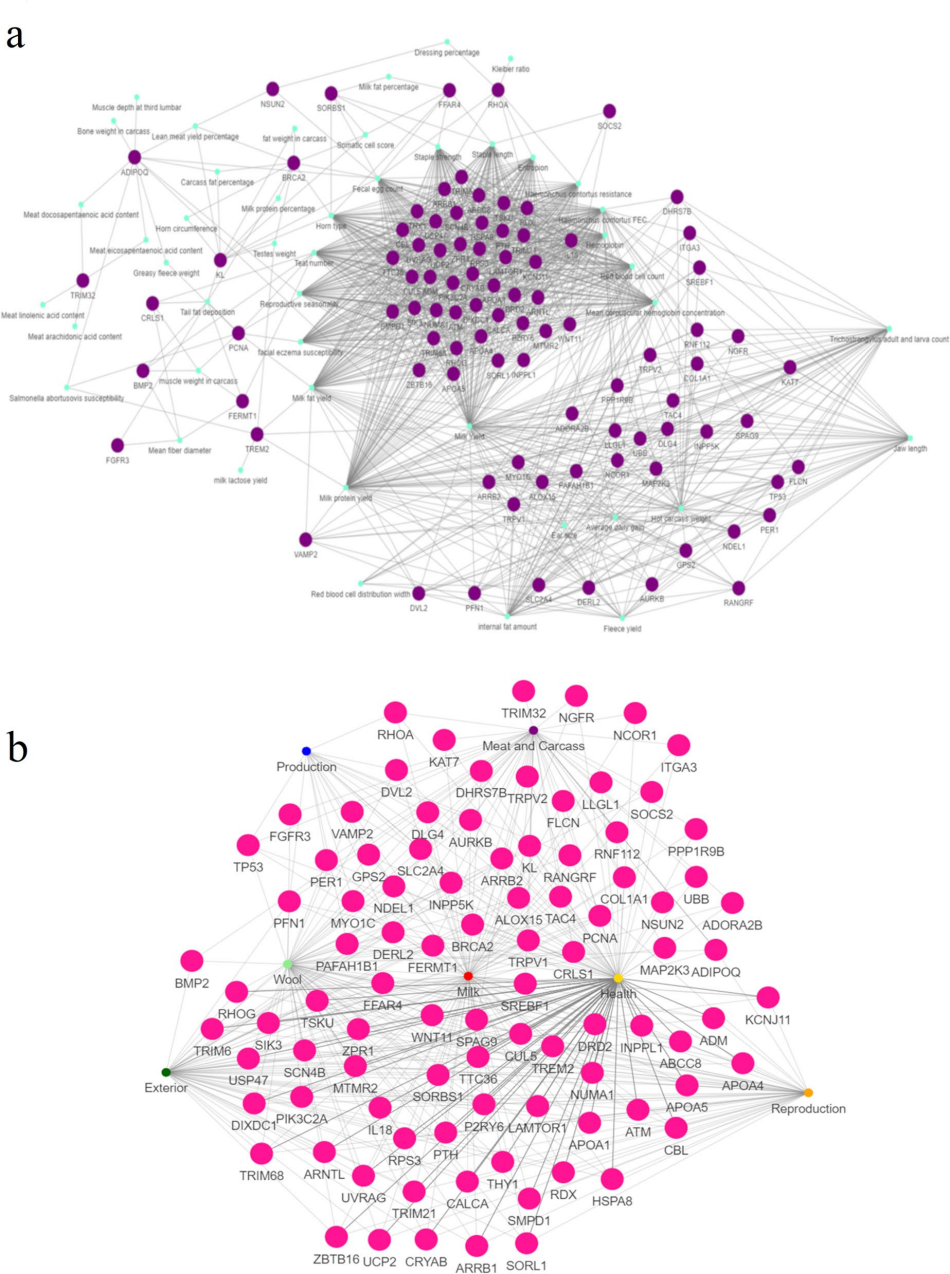
Interaction network composed by quantitative trait loci (QTL) and genes mapped in adaptation selective sweeps. **a** Interaction network for the hub genes (in green) identified in the gene ontology network harboring exclusively confirmed selective sweeps composed by more than 60% of adaptation (adapCSS) studies and the QTL traits (in purples) annotated. The edges between a QTL and a gene indicate that this gene is associated with the respective enriched QTL trait term; and **b** networks showing the relationship between the hub genes selected for adapCSS (in pink) and the different QTL trait types. The thickness of the edges represents the number of traits annotated for each QTL trait type and associated with the respective gene

**Fig. 4:**
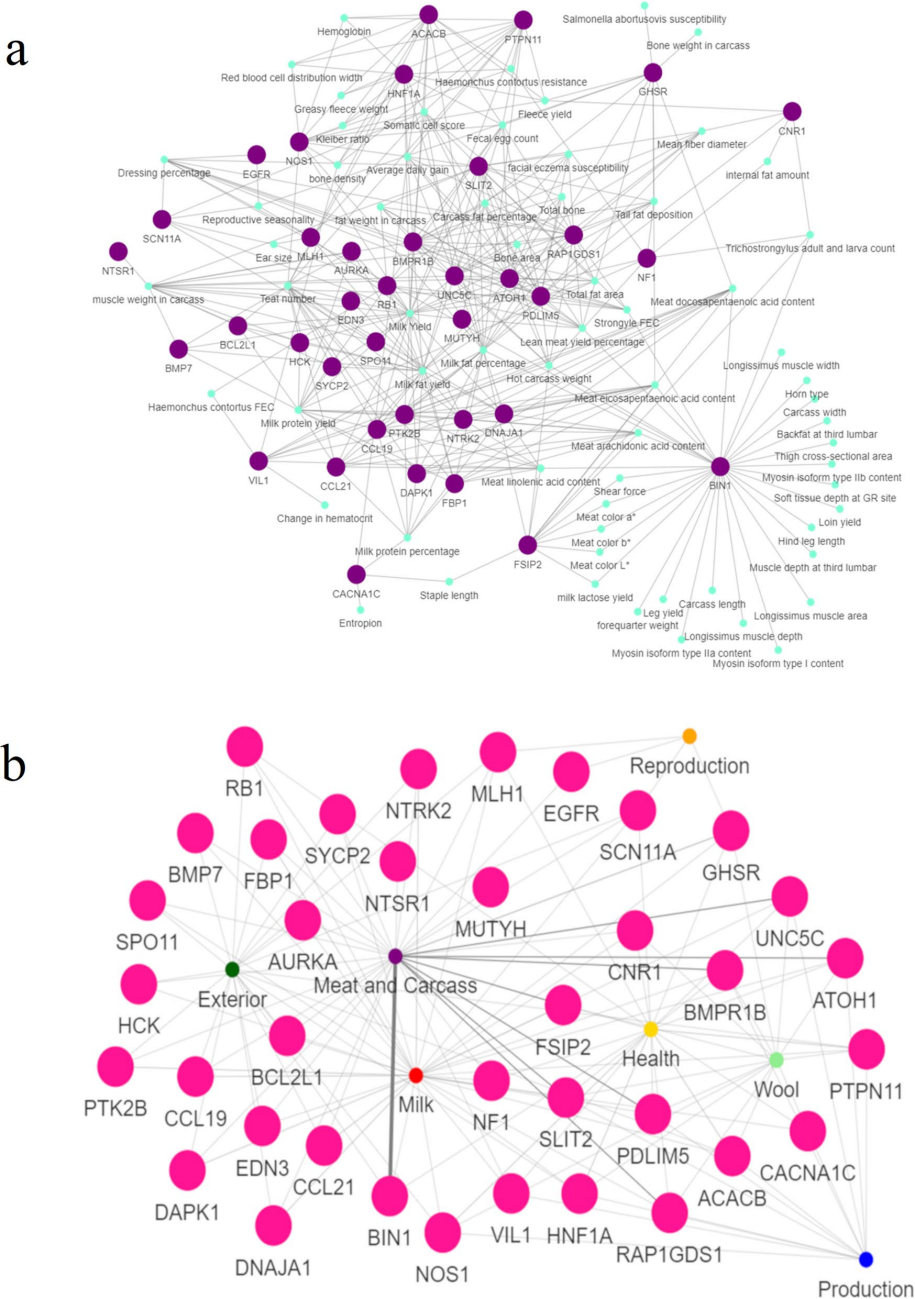
Interaction network composed by quantitative trait loci (QTL) and genes mapped in production selective sweeps. **a** Interaction network for the hub genes (in green) identified in the gene ontology network harboring exclusively confirmed selective sweeps composed by more than 60% of production (prodCSS) studies and the QTL traits (in purples) annotated. The edges between a QTL and a gene indicate that this gene is associated with the respective enriched QTL trait term; and **b** networks showing the relationship between the hub genes selected for prodCSS (in pink) and the different QTL trait types. The thickness of the edges represents the number of traits annotated for each QTL trait type and associated with the respective gene

**Fig. 5:**
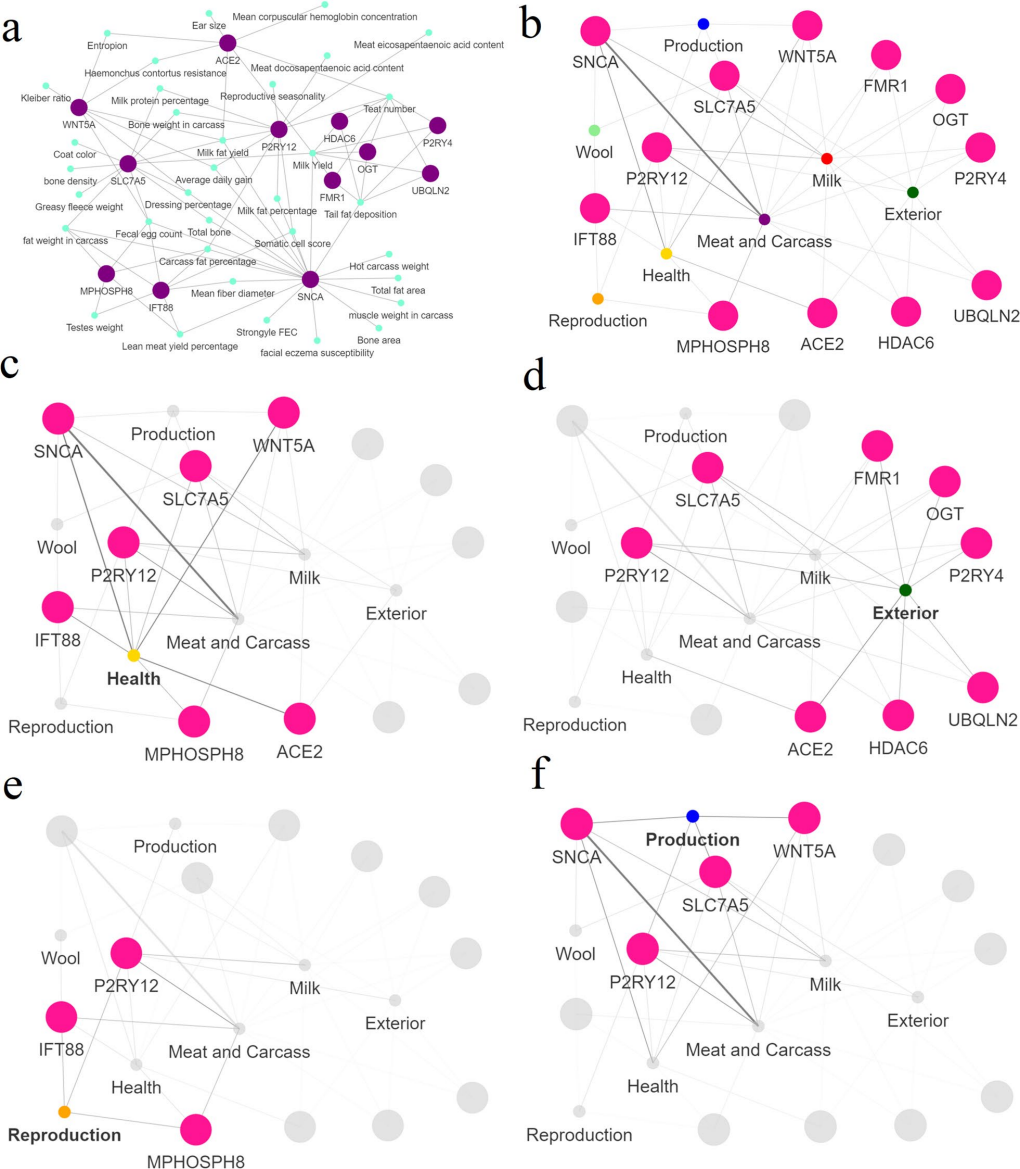
Interaction network composed by quantitative trait loci (QTL) and genes for the hub genes identified in the gene ontology network harboring both production (prodCSS) and adaptation (adapCSS) confirmed selective sweeps. **a** Networks showing the relationship between the hub genes (purple) selected and the different QTL terms (green). The edges between a QTL and a gene indicate that this gene is associated with the respective enriched QTL; **b**–**f** networks showing the relationship between the hub genes selected and the different QTL types. The thickness of the edges represents the number of traits annotated for each QTL type and associated with the respective gene. **b** Complete gene-QTL type network. **c** Network highlighting the direct connection between genes and health-related QTL. **d** Network highlighting the direct connection between genes and exterior-related QTL. **e** Network highlighting the direct connection between genes and reproduction-related QTL. **f** Network highlighting the direct connection between genes and production-related QTL

The betweenness of each gene in the network that was created based on the relationship between the genes harbouring CSS and the enriched GO terms was assessed for the three sets of enrichment results. The hub genes, defined as the genes with a betweenness above the 90% quantile, were selected for the enriched terms of genes harbouring only adapCSS (93 genes, quantile 90% threshold = 1084.64), genes harbouring only prodCSS (37 genes, quantile 90% threshold = 404.96), and genes harbouring both types of CSS (30 genes, quantile 90% threshold = 324.33). The betweenness values of each gene in the three networks are available in Additional file 10 Table S7. Genes harbouring CSS associated with enriched QTL terms were also considered associated with these QTL. Consequently, the relationships between genes and QTL were also represented as networks. The networks generated for the associations between the hub genes selected from the enriched GO term networks and the enriched QTL are shown in Figs. 3, 4 and 5.

The qualitative analysis of the network composed of the hub genes harbouring exclusively adapCSS (Fig. 3a) and the enriched QTL terms suggested the presence of a group of genes related to multiple health-related QTL traits (*Haemonchus contortus* resistance, facial eczema susceptibility, *Haemonchus contortus* FEC, entropion, haemoglobin, red blood cell count, faecal egg count, and mean corpuscular haemoglobin concentration). The same block of genes is also associated with morphological and reproduction-related QTL phenotypes, such as horn type, teat number, reproductive seasonality, and staple strength and length. A similar analysis of the distribution of the betweenness of these genes in this network suggested *ADIPOQ*, *PCNA*, *RHOA*, *TRIM32*, *TREM2*, *FFAR4*, *SORBS1*, *BRCA2,* and *KL* as potential hub genes (quantile 90% threshold = 153.97). *ADIPOQ,* which is the gene with the highest betweenness in this network, is associated with QTL for different contents of fatty acids in the meat as well as carcass fat percentage and reproductive seasonality. Interestingly, when the relationship between the selected hub genes for adapCSS and enriched QTL was analysed based on the QTL types (Fig. 3b), 88 out of the 93 genes were directly associated with health-related QTL. In addition, two clusters of genes harbouring adapCSS were observed, one directly associated with meat and carcass and production-related QTL terms (see Additional file 11 Figure S4) and another cluster composed of genes directly linked to reproduction-related QTL (see Additional file 11 Figure S4). In the meat and carcass cluster, it is relevant to highlight the presence of important genes for the control of lipid metabolism, such as *SREBF1*, *NCOR1*, *ALOX15*, *TRPV1*, and *TRPV2*.

The network created with the genes harbouring exclusively prodCSS (Fig. 4a, b) suggested that *BIN1*, *GHSR*, and *FSIP2* are potential hub genes for this network (quantile 90% threshold = 420.70). The *BIN1* gene showed a association with multiple carcass and meat-related QTL. The *GHSR* gene was directly linked to reproduction, wool, health, milk and meat- and carcass-related QTL. The *FSIP2* gene was linked to wool, health, milk and meat- and carcass-related QTL. The analysis of the networks composed of QTL term types indicated that almost all the selected genes are directly related to meat- and carcass-related QTL traits (see Additional file 12 Figure S5, excluding *NOS1*, *HNF1A*, *ACACB*, *CACNA1C*, and *PTPN11*) and milk-related QTL (see Additional file 12,Figure S5, excluding *NTSR1*, *MUTYH*, and *EGFR*). In addition, genes associated with wool-related QTL phenotypes (see Additional file 12 Figure S5) were also clustered close to health- and production-related QTL in the network analysis (Fig. 4b). In addition, exterior-related QTL were linked with a different cluster of genes compared to wool-, health- and production-related QTL (Fig. 4b and see Additional file 12 Figure S5). Reproduction-related QTL were associated with the *MLH1*, *EGFR*, *SCN11A*, and *GHSR* genes (Fig. 4b).

Finally, the network composed of hub genes from the enriched GO term networks harbouring both prodCSS and adapCSS and enriched QTL terms suggested that *SLC7A5* and *P2RX7* are hub genes of this network (quantile 90% threshold = 514.64). Important contributions to the network structure were also observed for the *SNCA*, *GRID2*, and *PKD2* genes. In the network, a direct connection between these genes and multiple QTL types can be observed, such as health QTL (strongyle FEC, facial eczema susceptibility, faecal egg count, and somatic cell score), meat and carcass QTL (lean meat yield percentage, hot carcass weight, carcass fat percentage, dressing percentage, total fat area, fat weight in carcass, tail fat deposition, and muscle weight in carcass), and milk-related QTL (milk fat percentage, milk fat yield, and milk yield) (Fig. 5a). The QTL type network (Fig. 5b) for these genes indicated a separation between genes linked to health-related QTL (Fig. 5c) and exterior-related QTL (Fig. 5d). In addition, a strong connection between meat and carcass-related QTL terms and the *GRID2*, *PKD2*, and *SNCA* genes was observed (Fig. 5e). The hub genes *SLC7A5* and *P2RX7* were also connected with production-related QTL that were clustered close to health- and wool-related QTL (Fig. 5f).

The description of enriched GO terms and QTL terms associated with these hub genes is shown in Additional file 13 Table S8. It is important to highlight that in spite of the selection of these potential hub genes, all the other genes included in the abovementioned networks are potential functional candidate genes for production and/or adaptation-related traits.

## Discussion

The combination of different sources of selection pressure results in the development of unique adaptative and production traits in livestock animals [6–8, 70–72]. The intense natural or artificial selection of favourable alleles for production and/or adaptation traits across the genome might reduce the genetic variability near those alleles due to the hitchhiking effect [73]. Selective sweeps are a specific type of genetic hitchhiking observed when directional selection is performed at a specific locus [74]. Here, the integration of studies that aim at identifying selective sweeps for adaptation and production traits across the sheep genome produces functional information about the specificities and similarities between these traits. It is relevant to highlight that among the 37 studies used to identify CSS, only two studies, Estrada-Reyes et al. [46] and McRae et al. [36], were also included in the SheepQTLdb [75]. Therefore, the QTL annotation and enrichment results obtained here can be interpreted as an independent validation of the association of these genomic regions with different production and adaptation traits.

### Genomic regions exclusively linked to adaptation CSS

In contrast to almost all livestock animals, a marked seasonality of breeding is observed in sheep, which can be caused by several factors, such as temperature, nutritional status, social interactions, and neuroendocrinal factors [76, 77]. Seasonality can be interpreted as an evolutionary response to environmentally challenging periods. Indeed, environmental factors such as heat and day length are described as affecting milk production in sheep [78–82]. Photoperiodism is one of the most important biological processes associated with the synchronization of the mammalian energy balance with environmental conditions [83]. The response to the light stimulus was present among the most enriched GO terms identified for genes harbouring exclusively adapCSS. Among the genes associated with the control and regulation of the circadian clock, such as *TP53* [84], *GNAQ* [85], *DRD2* [86], *USP2* [87], and *PER1* [88] and photoreceptor function *AIPL1* [89] and *GNAT1* [90], stand out as relevant candidate genes for signatures of selection associated with adaptation to seasonality in sheep. The other two functionally enriched GO terms observed for the list of genes harbouring exclusively adapCSS were growth and animal organ maturation. The ability of an animal to grow and properly develop in its environment is a crucial characteristic of its adaptation. Three genes were shared between these two terms (*EXT1*, *FGFR3*, and *RHOA*). The *EXT1* gene encodes a protein responsible for the elongation step of heparan sulfate biosynthesis, which is associated with the regulation of the development of the brain [91, 92] and bones [93, 94], as well as the gastrulation process [95]. FGFR3 is a receptor tyrosine kinase and acts negatively to regulate bone growth [96]. Finally, RHOA (a hub gene in the current study) is a member of the Rho family of small GTPases and belongs to the RhoA/ROCK pathway responsible for neuronal migration, dendrite development, and axonal extension [97]. In addition, *RHOA* was associated with chronic hypoxic foetal and adult sheep, suggesting an important role of RhoA pathways in hypertension control in newborns. In sheep, pulmonary hypertension in the newborn is a critical condition in breeds located at high altitudes [98]. The presence of CSS, including the *RHOA* gene, might suggest the presence of variants in this gene that contribute to resistance to pulmonary hypertension.

Among the enriched GO terms and QTL terms associated with the genes harbouring exclusively adapCSS, an association with lipid metabolism was observed. For example, the main hub gene identified in the networks composed of GO terms and QTL terms was the *ADIPOQ* gene. This gene encodes the hormone adiponectin, which is responsible for acting in the hypothalamus, stimulating food intake [99]. In addition, adiponectin acts in triglyceride hydrolysis, fatty acid decomposition, fatty acid oxidation and lipid synthesis [100, 101].

In livestock species, variants in the *ADIPOQ* gene are associated with marbling [102, 103], body measurements [104, 105], and growth and carcass traits [106]. It is crucial to recall that lipid metabolism plays an important role in sheep adaptation to extreme environments [107]. In addition, seasonality and adiposity in the body are associated; for instance, seasonal changes in body weight and fat percentage are observed frequently [108]. Other relevant candidate genes for lipid metabolism that are widely associated with meat and milk production traits in livestock species, such as *SCD* [109–113] and *SREBF1* [114–118], were also identified among the genes harbouring exclusively adapCSS. Genes associated with lipid metabolism and adaptation are often associated with thermoregulation in mammals [119–121]. In total, 24 genes harbouring only adapCSS were associated with the GO term adaptive thermogenesis. The abovementioned genes *ADIPOQ* [122], *SCD* [123] and *SREBF1* [124] are closely related to the control of thermogenesis and energy homeostasis. In addition, other genes involved in important processes associated with brown and white fat physiology were identified as exclusively harbouring adapCSS, such as *ELOVL3* [125, 126], *FLCN* [127], *TRPV1* [128], *TRPV2* [129], *OXT* [130], *IL18* [131], *UCP2* [132, 133], and *UCP3* [132, 134]. It is important to highlight the association of several genes harbouring adapCSS with parasite resistance-related enriched QTL (i.e., *Salmonella abortusovis* susceptibility, *Haemonchus contortus* FEC, facial eczema susceptibility, and *Haemonchus contortus* resistance). Interestingly, *ADIPOQ*, *SREBF1*, and *FLCN* were observed among those genes associated with these enriched QTL terms. The formation of lipid droplets which is one of the different functions performed by these genes [135–137], is a process that is reported to play an important role in the interaction between hosts and pathogens [105], which suggests a potential role of these genes (and others identified in the current study) in pathogen resistance in sheep. Among the other hub genes identified as harbouring exclusively prodCSS, the *FFAR4* gene was previously associated with the regulation of glucose homeostasis, adiposity, gastrointestinal peptide secretion, and taste preference [138].

Another relevant result was obtained by evaluating the most-enriched QTL term observed for adapCSS, i.e. horn type. All the adapCSS associated with the QTL for horn type (and horn circumference) are mapped on chromosome 10. In addition, the majority of these adapCSS are mapped to a region comprising the coordinates of the *RXFP2* gene, a gene involved in the development of sexual characteristics in humans and mice [139, 140]. A 1.8-kb insertion in the 3′-UTR of this gene was previously associated with polledness in sheep [141, 142]. In addition, *RXFP2* is suggested to act in the development of unique horn phenotypes as a response to semiferalization (the partial animal reversion to life in nature, with human artificial selection no longer dominant) [143].

These results reinforce the association of the adapCSS identified in the current study with relevant biological processes that are directly associated with adaptation to environmental and physiological challenges in sheep breeds. In addition, the faster response and recovery to challenges that disturb animal health, welfare and production are the basis of selection for more resilient animals [20, 25].

### Genomic regions harbouring production CSS exclusively

Interestingly, reproduction and feeding behaviour-related terms were among the most enriched terms associated with the genes harbouring exclusively prodCSS [112–114]. Important functional candidate genes for reproductive traits, such as *ADCY10* [144], *B4GALT1* [145], *PGR* [146], *TBX3* [147], *SPACA1* [148], *SPATA16* [149], and *SYCP2* [150], and feeding behaviours, such as *CNTFR* [151], *DMBX1* [152], *NTRK2* [153], *PYY* [154], and *RMI1* [155], were observed among those associated with these enriched terms. In addition, a link was observed between reproduction and feeding behaviour by the presence of the genes *CNR1* and *GHSR* (a hub gene in the current study) in both terms [127]. The expression of *CNR1* in cortical neurons was shown to be increased after fasting, suggesting a role in feeding [156]. Regarding the association with reproductive traits, the action of *CNR1* is associated with important sperm functions and testicular development [157]. It is important to highlight that *CNR1* was also classified as a hub gene in the network analysis composed of the genes harbouring exclusively prodCSS and enriched GO terms. Similarly, the *GHSR* gene, which encodes the ghrelin receptor, acts in both processes. Ghrelin is a gastrointestinal peptide hormone involved in several biological processes, such as gut motility, gastric acid secretion, sleep and wake rhythm, taste sensation, glucose metabolism, and regulation of food intake [158–160]. Interestingly, genetic polymorphisms in *GHSR* were previously associated with body composition and growth in chickens and cattle [161–163]. In addition, ghrelin is suggested to be a regulator of the gonadotropic axis with a predominant negative effect through a signal of energy deficit [164]. In sheep, *GHSR* expression was detected in the adult testis and ovary with a significant effect of season (photoperiod) on its expression in the testis [165].

Heat stress resistance is an important, economically relevant trait in sheep due to its impact on several other traits, such as wool, milk production, immunity, and reproductive performance [166–170]. In total, 17 genes (*ADRB2*, *ARPP21*, *CPB2*, *DNAJA1*, *DNAJA2*, *EIF2AK4*, *FBP1*, *HTR2A*, *MSTN*, *NF1*, *NOS1*, *NTSR1*, *POLR2D*, *SCN11A*, *SLC52A3*, *STAC*, and *TRPV4*) identified in regions exclusively harbouring prodCSS were associated with the enriched GO term “response to temperature stimulus”. The search for genetic markers responsible for better resilience to heat stress is a key step for improving small ruminant productivity, welfare and health [171]. The genes *BIN1*, *GHSR*, and *FSIP2* were above the quantile 90% for the betweenness distribution for all genes included in the network composed by genes and enriched QTL. The functions associated with the *GHSR* were previously discussed in this subsection. The *BIN1* gene encodes the protein amphiphysin, an important regulator of cell muscle differentiation and maturation [172–175]. Consequently, polymorphisms in *BIN1* could be associated with meat and carcass traits in sheep breeds. *FSIP2* plays a crucial role in acrosome development and, consequently, in male fertility [176]. Although it was not defined as a hub gene by the criteria defined in the current study for the gene-QTL network, the *SLIT2* gene showed an association pattern with milk-related QTL in the analysed networks. The action of *SLIT2* in the mammary gland is associated with stem cell self-renewal and the generation of tubular bilayers during ductal morphogenesis [177, 178].

### Genomic regions harbouring CSS for production and adaptation

Certain genetic loci can concurrently influence multiple complex traits, a phenomenon known as pleiotropy [179]. Pleiotropy may lead to the inadvertent selection of undesirable hitchhiking effects. The identification of genomic regions and/or variants associated with pleiotropic effects has the potential to improve the development of multivariate trait analysis and, subsequently, improve selection indices, resulting in greater genetic enhancement [180–183]. However, it is important to highlight that pleiotropy cannot be exclusively the cause of genetic correlation between traits, as linkage and gametic disequilibrium between loci can also strongly contribute to this phenomenon [155].

In the current study, several genes harbouring prodCSS and adapCSS were identified. The two most enriched GO terms for this list of genes were associated with purinergic nucleotide receptor activity. The purinergic receptors can be classified as ionotropic (P2X) and metabotropic (P2Y) and act over a multitude of biological processes; however, their actions over neurotransmitter release, synaptic plasticity, and cellular proliferation, differentiation, degeneration and regeneration stand out [184]. Among the genes associated with purinergic receptor activity identified here, both ionotropic (*P2RX2*, *P2RX4*, and *P2RX7*) and metabotropic (*P2RY12*, *P2RY13*, *P2RY14*, and *P2RY4*) genes were identified. The *P2RX7* gene, which plays a crucial role in the modulation of inflammation and pain [185], was considered a hub gene in the network composed of genes and enriched QTL in the current study. In addition, genes encoding G-protein coupled receptors (*GPR34* and *GPR87*) and a Ca^2+^-binding protein (*NECAB2*) were also associated with these enriched terms. The abovementioned genes are involved in, among other processes, the regulation of the immune system and the response to pain [186–190]. The activation of the immune response and sensitivity to stressful stimuli, such as pain, are at the interface between disease resistance and productivity. The improvement in immunological response and resistance is suggested to occur at the cost of productivity caused by the redirection of nutrient use [191]. In addition, genetic variations observed in this trade-off between higher immunity and productivity might be explained by variations in the sensitivity of the stimulus to trigger the immune system and the number of signals generated by its activation [191]. In addition, in cattle, *P2RY12*, *P2RY14*, and *GPR87* were considered as functional candidate genes for 305-day milk yield [192], reinforcing the potential role of these genes in productivity. Indeed, *P2RY12* was associated with all types of QTL, excluding wool-related QTL, in the network composed of hub genes and enriched QTL.

Terms related to acetyl-CoA were also identified as enriched for genes harbouring both prodCSS and adapCSS (*ACSS1*, *MLYCD*, *MVD*, *NUDT7*, *PDHA1*, *PDHB*, *PDK3*, and *TDO2*). In sheep, a decrease in fatty acid synthesis in the adipose tissue during lactation is observed due to a decrease in total acetyl-CoA carboxylase activity and the proportion of the enzyme in the active state [193]. The *ACSS1* gene, which encodes an acetyl-CoA synthase, was identified among the genes associated with acetyl-CoA metabolism. This gene is associated with a response to metabolic stress [194] through acetate-mediated epigenetic regulation, which can also induce fatty acid synthesis [195]. Furthermore, the *ACSS1* gene has been previously associated with lipid metabolism in relation to milk and meat composition in cattle and sheep [196–200]. Another gene associated with acetyl-CoA metabolism in the current study, *MVD*, was previously reported in selective sweeps for adaptation and productivity in different cattle breeds [201–203]. This gene encodes the enzyme mevalonate pyrophosphate decarboxylase, which is responsible for the conversion of mevalonate pyrophosphate into isopentenyl pyrophosphate during one of the first stages of cholesterol biosynthesis [204].

The coat colour QTL term was enriched only for adapCSS in the current study. However, adapCSS and prodCSS were associated with the coat colour QTL trait term during the annotation process. The majority of the CSS associated with these QTL are mapped in the region of chromosome 14 that carries the *MC1R* gene, which harbours both prodCSS and adapCSS. Mutations in *MC1R* are associated with the determination of coat colour in different sheep breeds [205, 206]. In addition, a region on chromosome 1, comprising the *RUNX1* gene, was associated with coat colour QTL for both adapCSS and prodCSS. *RUNX1* encodes a transcription factor associated with the development of hair and other skin appendages [207, 208]. Consequently, *RUNX1* emerges as a candidate for coat colour in sheep breeds. In addition, it is important to mention that the region comprising the *ASIP1* gene, another gene traditionally associated with coat colour in sheep [209, 210], was observed among the CSS identified with 50 and 60% of production and adaptation studies. These CSS were not included in the downstream functional analyses. However, the link suggested by our integrative analysis between coat colour and adapCSS highlights the known association between coat colour and adaptation to challenging environmental conditions, such as heat stress [211]. In addition, in some breeds, coat colour has an antagonistic effect on size and fitness [212]. Consequently, coat colour is a candidate phenotype to understand the relationship between productivity and adaptability in sheep breeds.

One other gene highlighted in the network between enriched GO terms and QTL trait terms derived from the genes associated with both prod-CSS and adaptCSS was *SNCA.* This gene encodes alpha-synuclein, a protein highly expressed in the presynaptic terminals of the central nervous system, associated with neurodegenerative disorders [213]. In sheep and goats, alpha-synuclein accumulates in their brains during scrapie infection, suggesting that perturbations in alpha-synuclein metabolism might play a role in prion infection [214]. Scrapie is a relevant health issue due to its neurodegenerative, progressive and lethal characteristics in sheep and goats, resulting in substantial efforts to reduce the disease incidence by selecting more resistant animals [215, 216]. The regulatory process of alpha-synuclein seems to play a crucial role in the brain inflammatory response through the modulation of lipid metabolism in the brain [217]. A link between *SNCA* and health-, meat- and carcass-related QTL trait terms was observed here. Genetic variants that map to the genomic regions harbouring the *SNCA* gene in pigs and cattle have been previously associated with backfat thickness [218] and milk somatic cell count [219], respectively. There is no direct evidence of the role of *SNCA* in production traits in livestock species. However, some studies suggest the action of alpha-synuclein as a glucoregulator in adipose tissue and skeletal muscle [220, 221], which might help explain a potential role in production-related traits in sheep. Another hypothesis is a potential hitchhiking effect observed between the *SNCA* locus and the *NCAPG*-*LCORL* locus. These two genomic regions are 2.31 Mb apart (based on the ARS-UI_Ramb_V2.0 reference genome). The *NCAPG*-*LCORL* locus is one of the most relevant loci associated with pleiotropic effects in livestock species, with associations reported for height, body weight, feed intake, gain, age at puberty, and meat and carcass traits [222–227].

## Conclusions

Production and adaptation are intrinsically related processes in livestock species due to the intensive selective pressures for higher productivity levels to which these animals are subjected, driving the development of unique adaptive and production traits. Based on the identification of CSS associated with production and adaptation in sheep breeds, the present study pinpoints functional candidate genes for productivity and adaptability and candidate genes with the potential to simultaneously regulate both classes of traits. Intriguingly, a relevant role of lipid metabolism arose among the functional candidate genes that were identified in regions exclusively associated with adaptation or production. In addition, on the one hand, for adaptation-related regions, relevant functional candidate genes for the control of seasonality, circadian rhythm, and thermoregulation were observed. On the other hand, for production regions, genes associated with the control of feeding behaviour, reproduction, and cellular differentiation stand out as relevant functional candidates. However, it is important to highlight that the selection signals evaluated here are the result of directional selection and do not reflect signals subjected to balancing selection. In addition, selective sweeps on the X chromosome and interactions between mitogenome-autosomes were not investigated due to the absence of such results in the evaluated manuscripts. Consequently, not all sources and/or signals of selective sweeps were analysed here. The results obtained here help elucidate the genetic relationship between productivity and adaptability in sheep breeds. In addition, a series of functionally-relevant candidate genes are provided, which may help improve the fine-mapping of genomic regions for further studies aiming at the identification of causal variants associated with higher productivity and/or adaptability and resiliency in sheep.

### Supplementary Information

Supplementary Material 1: Figure S1. Workflow for the selection of studies to be included in the pipeline for the identification of confirmed selective sweeps associated with production or adaptation traits.

Supplementary Material 2: Table S1. Studies reporting selection signatures for production or adaptation traits across the sheep genome retrieved from Pubmed initially considered for this integrative review.

Supplementary Material 3: Table S2. Genomic coordinates of all selective sweeps reported in the 37 studies retained for the identification of confirmed selective sweeps.

Supplementary Material 4: Table S3. Confirmed selective sweeps (CSS) identified through the integration of the results obtained in the 37 individual studies evaluated in the present study.

Supplementary Material 5: Figure S2. Distribution of the percentage of production (A) and adaptation (B) studies associated with each confirmed selective sweeps. The bar in red are above the threshold (60%) defined to classify a CSS as production or adaptation CSS.

Supplementary Material 6: Table S4. Genes annotated within the coordinates of the confirmed selective sweeps (CSS).

Supplementary Material 7: Table S5. Results of quantitative trait loci (QTL) enrichment analysis performed for the traits annotated within the confirmed selective sweeps (CSS) coordinates for production and adaptation traits in the SheepQTLdb.

Supplementary Material 8: Table S6. Results of Gene Ontology (GO) terms enrichment analysis for the genes harboring production and adaptation confirmed selective sweeps.

Supplementary Material 9: Figure S3. Network composed by genes (in purple) and enriched Gene Ontology terms (in green) associated with lipid metabolism identified for the list of genes harboring exclusively confirmed selective sweeps composed by more than 60% of adaptation studies.

Supplementary Material 10: Table S7. Betweenness values estimated for each gene present in the enriched gene ontology network network composed by genes harboring exclusively production and adaptation confirmed selective sweeps (CSS) and genes harboring both types of CSS.

Supplementary Material 11: Figure S4. Interaction network composed by Quantitative trait loci (QTL) classes and genes (pink) for the hub genes identified in the gene ontology network harboring exclusively confirmed selective sweeps composed by more than 60% of adaptation (adapCSS) studies. The edges between a QTL and a gene indicate that this gene is associated with the respective QTL class. A) Network highlighting the direct connection between genes and health-related QTL trait terms. B) Network highlighting the direct connection between genes and meat and carcass-related QTL trait terms. C) Network highlighting the direct connection between genes and production-related QTL trait terms. D) Network highlighting the direct connection between genes and reproduction-related QTL trait terms.

Supplementary Material 12: Figure S5. Interaction network composed by Quantitative trait loci (QTL) classes and genes (pink) for the hub genes identified in the gene ontology network harboring exclusively confirmed selective sweeps composed by more than 60% of production (prodCSS) studies. The edges between a QTL and a gene indicate that this gene is associated with the respective QTL class. A) Network highlighting the direct connection between genes and meat and carcass-related QTL. B) Network highlighting the direct connection between genes and milk-related QTL. C) Network highlighting the direct connection between genes and wool-related QTL. D) Network highlighting the direct connection between genes and production-related QTL.

Supplementary Material 13: Table S8. Association between the selected hub genes for production and adaptation (or shared) confirmed selective sweeps with enriched gene ontology terms and quantitative trait loci annotated in the SheepQTLdb.

## Data Availability

All the data used in the current study is available in the manuscript or supplementary information.
