# Supplementary material for: Integration of selective sweeps across the sheep genome: understanding the relationship between production and adaptation traits
Source: Genet Sel Evol. 2024 May 21;56:40. doi: 10.1186/s12711-024-00910-w (PMC11106937; doi:10.1186/s12711-024-00910-w)
Supplement: Supplementary file 1 — Supplementary Material 1: Figure S1. Workflow for the selection of studies to be included in the pipeline for the identification of confirmed selective sweeps associated with production or adaptation traits. [file 12711_2024_910_MOESM1_ESM.docx]

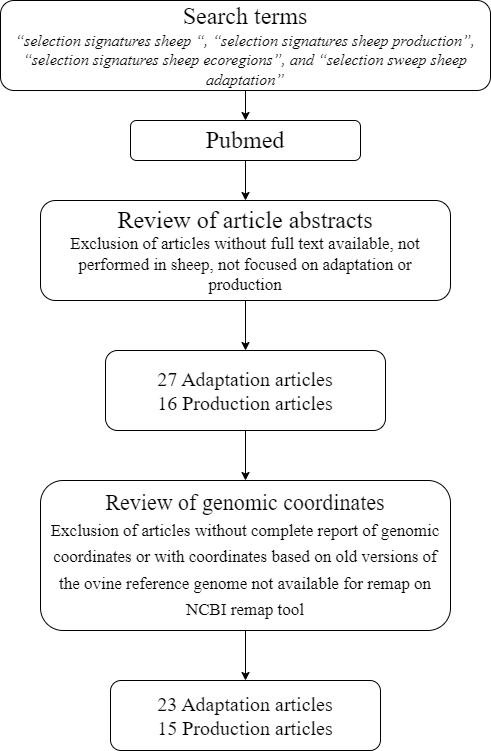


Figure S1: Workflow for the selection of studies to be included in the pipeline for the identification of confirmed selective sweeps associated with production or adaptation traits.
