# Supplementary material for: Integration of selective sweeps across the sheep genome: understanding the relationship between production and adaptation traits
Source: Genet Sel Evol. 2024 May 21;56:40. doi: 10.1186/s12711-024-00910-w (PMC11106937; doi:10.1186/s12711-024-00910-w)
Supplement: Supplementary file 5 — Supplementary Material 5: Figure S2. Distribution of the percentage of production (A) and adaptation (B) studies associated with each confirmed selective sweeps. The bar in red are above the threshold (60%) defined to classify a CSS as production or adaptation CSS. [file 12711_2024_910_MOESM5_ESM.docx]

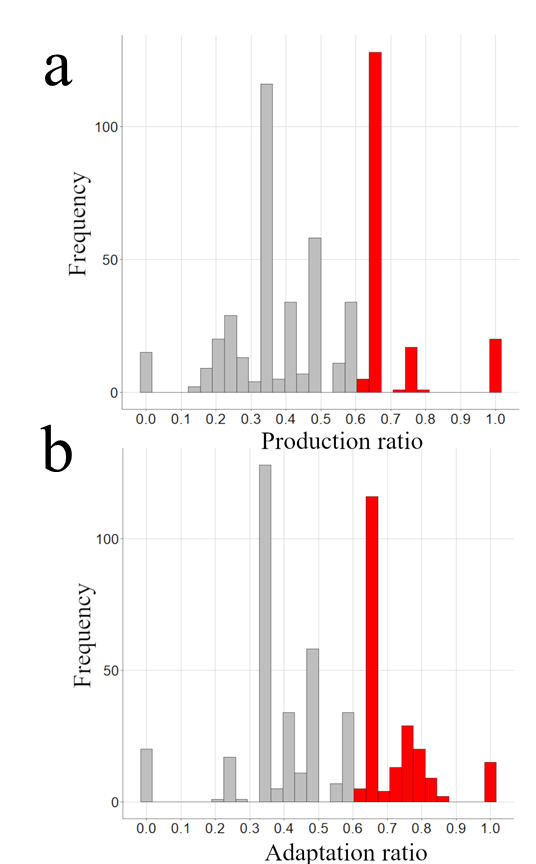


Figure S2: Distribution of the percentage of production (A) and adaptation (B) studies associated with each confirmed selective sweeps. The bar in red are above the threshold (60%) defined to classify a CSS as production or adaptation CSS.
