# Supplementary material for: Integration of selective sweeps across the sheep genome: understanding the relationship between production and adaptation traits
Source: Genet Sel Evol. 2024 May 21;56:40. doi: 10.1186/s12711-024-00910-w (PMC11106937; doi:10.1186/s12711-024-00910-w)
Supplement: Supplementary file 9 — Supplementary Material 9: Figure S3. Network composed by genes (in purple) and enriched Gene Ontology terms (in green) associated with lipid metabolism identified for the list of genes harboring exclusively confirmed selective sweeps composed by more than 60% of adaptation studies. [file 12711_2024_910_MOESM9_ESM.docx]

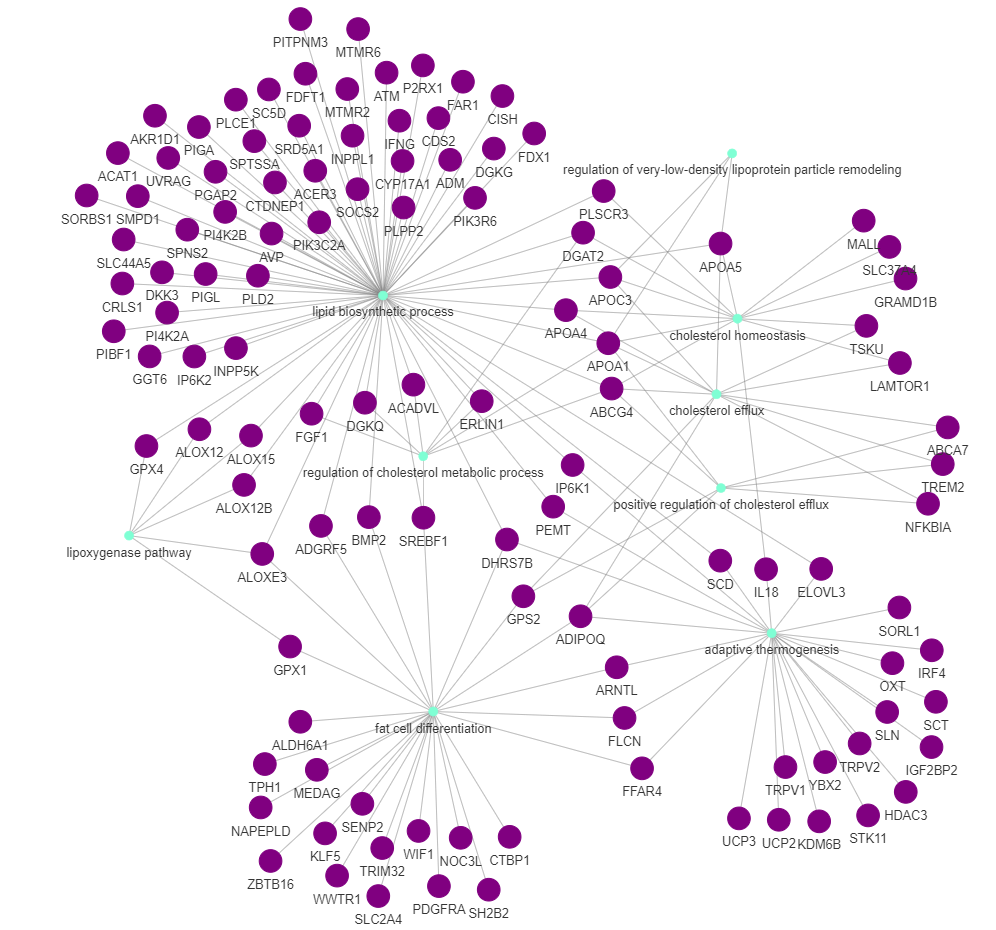


Figure S3: Network composed by genes (in purple) and enriched Gene Ontology terms (in green) associated with lipid metabolism identified for the list of genes harboring exclusively confirmed selective sweeps composed by more than 60% of adaptation studies.
