# Supplementary material for: Integration of selective sweeps across the sheep genome: understanding the relationship between production and adaptation traits
Source: Genet Sel Evol. 2024 May 21;56:40. doi: 10.1186/s12711-024-00910-w (PMC11106937; doi:10.1186/s12711-024-00910-w)
Supplement: Supplementary file 11 — Supplementary Material 11: Figure S4. Interaction network composed by Quantitative trait loci (QTL) classes and genes (pink) for the hub genes identified in the gene ontology network harboring exclusively confirmed selective sweeps composed by more than 60% of adaptation (adapCSS) studies. The edges between a QTL and a gene indicate that this gene is associated with the respective QTL class. A) Network highlighting the direct connection between genes and health-related QTL trait terms. B) Network highlighting the direct connection between genes and meat and carcass-related QTL trait terms. C) Network highlighting the direct connection between genes and production-related QTL trait terms. D) Network highlighting the direct connection between genes and reproduction-related QTL trait terms. [file 12711_2024_910_MOESM11_ESM.docx]

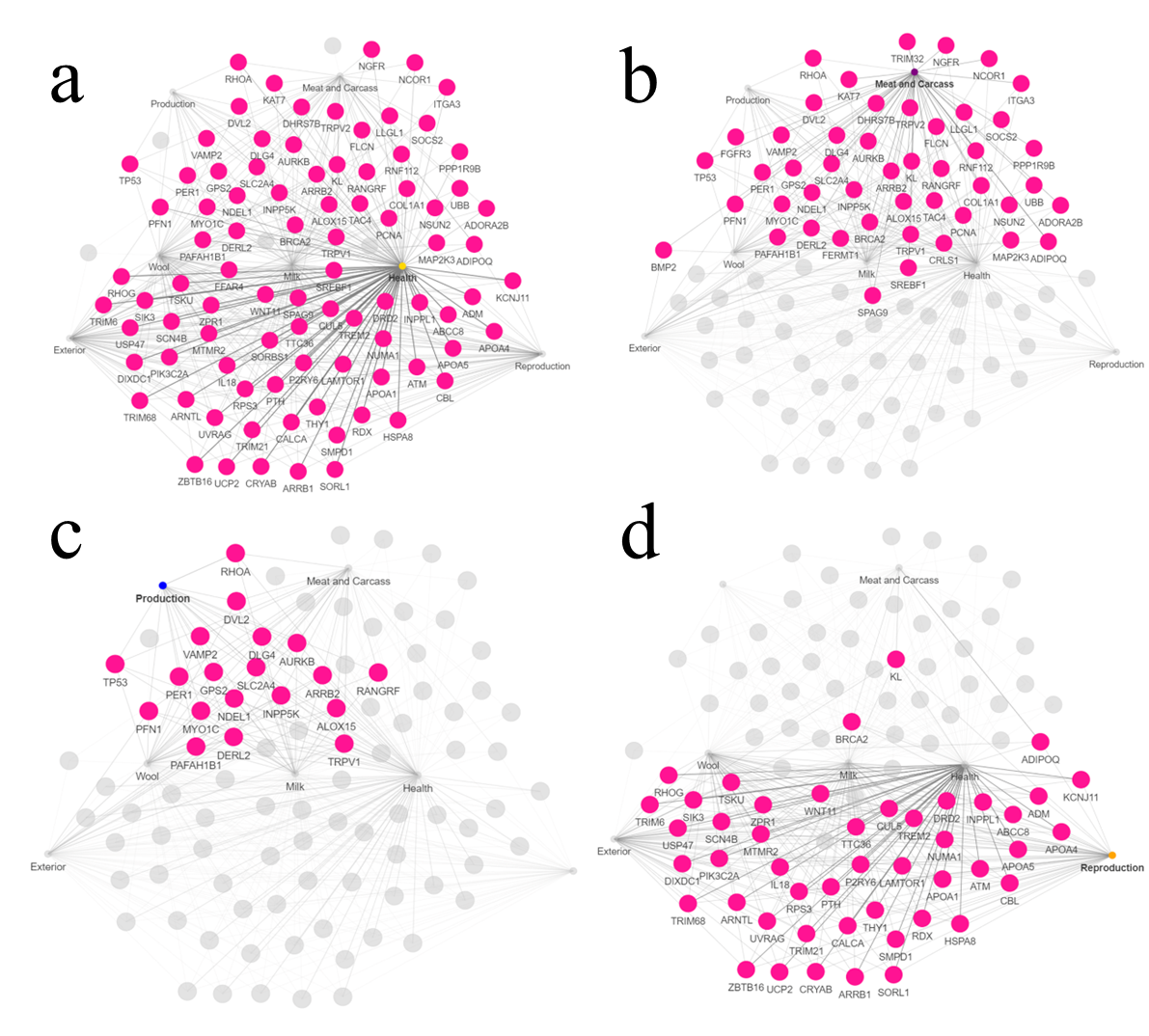


Figure S4: Interaction network composed by Quantitative trait loci (QTL) classes and genes (pink) for the hub genes identified in the gene ontology network harboring exclusively confirmed selective sweeps composed by more than 60% of adaptation (adapCSS) studies. The edges between a QTL and a gene indicate that this gene is associated with the respective QTL class. A) Network highlighting the direct connection between genes and health-related QTL trait terms. B) Network highlighting the direct connection between genes and meat and carcass-related QTL trait terms. C) Network highlighting the direct connection between genes and production-related QTL trait terms. D) Network highlighting the direct connection between genes and reproduction-related QTL trait terms.
