# Supplementary material for: Integration of selective sweeps across the sheep genome: understanding the relationship between production and adaptation traits
Source: Genet Sel Evol. 2024 May 21;56:40. doi: 10.1186/s12711-024-00910-w (PMC11106937; doi:10.1186/s12711-024-00910-w)
Supplement: Supplementary file 12 — Supplementary Material 12: Figure S5. Interaction network composed by Quantitative trait loci (QTL) classes and genes (pink) for the hub genes identified in the gene ontology network harboring exclusively confirmed selective sweeps composed by more than 60% of production (prodCSS) studies. The edges between a QTL and a gene indicate that this gene is associated with the respective QTL class. A) Network highlighting the direct connection between genes and meat and carcass-related QTL. B) Network highlighting the direct connection between genes and milk-related QTL. C) Network highlighting the direct connection between genes and wool-related QTL. D) Network highlighting the direct connection between genes and production-related QTL. [file 12711_2024_910_MOESM12_ESM.docx]

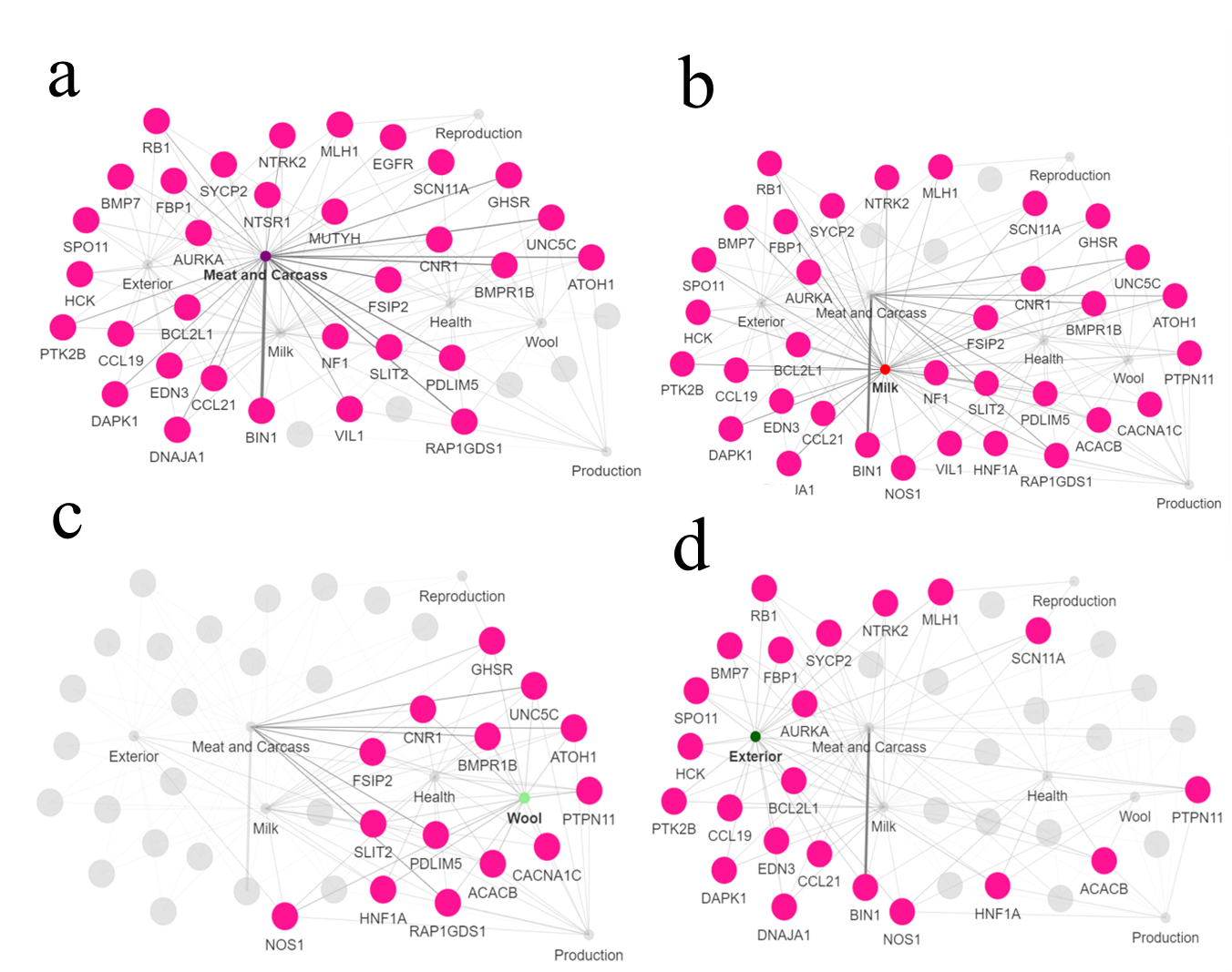


Figure S5: Interaction network composed by Quantitative trait loci (QTL) classes and genes (pink) for the hub genes identified in the gene ontology network harboring exclusively confirmed selective sweeps composed by more than 60% of production (prodCSS) studies. The edges between a QTL and a gene indicate that this gene is associated with the respective QTL class. A) Network highlighting the direct connection between genes and meat and carcass-related QTLs. B) Network highlighting the direct connection between genes and milk-related QTLs. C) Network highlighting the direct connection between genes and wool-related QTLs. D) Network highlighting the direct connection between genes and production-related QTLs.
